# Factors associated with functional disability in patients with acute stroke excluded from alteplase administration due to minor non-disabling neurological deficits

**DOI:** 10.3389/fneur.2022.1062721

**Published:** 2022-12-22

**Authors:** Yong Jin Kim, Seok Hong Choi, Tae Young Kim, Hyeon-Mi Park, Dong Jin Shin, Dong Hoon Shin

**Affiliations:** Department of Neurology, Gachon University College of Medicine, Incheon, South Korea

**Keywords:** alteplase, acute ischemic stroke, minor, hyperacute treatment, functional outcome after acute stroke

## Abstract

**Background:**

Although the PRISMS study did not demonstrate the benefit of intravenous alteplase administration in patients with mild stroke within 3 h, about 30% of patients presenting with mild symptoms showed unfavorable functional outcomes. We investigated the factors predictive of functional disability at 90 days in patients who were excluded from alteplase administration due to the National Institutes of Health Stroke Scale (NIHSS) scores of 0–5 and a score between 0 and 2 for each NIHSS score item.

**Methods:**

All patients were diagnosed with acute ischemic stroke or transient ischemic attack within 4.5 h of admission to a tertiary hospital and did not receive alteplase due to a minor stroke between January 2013 and December 2020. Radiological data and clinical information were collected, including baseline and discharge NIHSS scores and modified Rankin Scale (mRS) scores at 90 days. Early neurological deterioration (END) was defined as an increase of two or more NIHSS scores. We defined moderate motor weakness as a NIHSS limb motor score of more than 3 and defined a favorable outcome as a mRS score at 90 days that was 0 or 1.

**Results:**

During the investigation period, 400 patients did not receive alteplase. END occurred significantly more frequently in patients with large artery disease (LAD) than in those with other TOAST classifications. In the multivariate regression analysis, NIHSS per 1-point increase, presenting as moderate motor weakness, and LAD were independent predictors of poor functional outcome (OR, 1.811 NIHSS per 1-point increase; 95% confidence interval [CI], 1.503–2.182; *P* < 0.0001; OR, 2.173 moderate motor weakness; 95% CI 1.028–4.595; *P* = 0.042; OR, 2.033 LAD; 95% CI 1.099–3.762; *P* = 0.024, respectively).

**Conclusion:**

Moderate motor weakness presentation and LAD may be important factors associated with poor functional outcomes in patients with acute stroke excluded from alteplase administration due to mild symptoms.

## 1. Introduction

Intravenous (IV) thrombolytic treatment with alteplase, initiated within 4.5 h after the onset of symptoms, has been shown to reduce patient disability compared with placebo (1, 2). However, the effects of alteplase in patients with mild symptoms are unclear because many clinical trials involving the use of alteplase have excluded these patients (3–5).

A mild stroke symptom is the most common cause for the exclusion of thrombolytic treatment (6). However, many of these patients have recurrent events or disabilities despite mild symptoms at the initial presentation. Prospective data suggest that 30% of patients with the National Institutes of Health Stroke Scale (NIHSS) scores 0–3 or transient ischemic attack (TIA) have a functional disability 90 days after stroke (7). Therefore, to avoid disability, the number of IV alteplase administrations for patients with mild symptoms has increased (8).

The Phase IIIb, Double-Blind, Multicenter Study to Evaluate the Efficacy and Safety of Alteplase in Patients with Mild Stroke: Rapidly Improving Symptoms and Minor Neurologic Deficits (PRISMS) trial tested the efficacy and safety of IV alteplase in patients with NIHSS scores 0–5 and non-disabling deficits; however, IV alteplase administration was not beneficial within 3 h of onset (9). Based on the finding of this trial, IV alteplase is not recommended for patients with mild stroke in the current AHA/ASA guidelines (strength of recommendation class III [No benefit]; quality of evidence level B-R [Randomized]) (10). Although the PRISMS study did not demonstrate an effect in patients with mild stroke, 6–15% of patients with acute ischemic stroke presenting with minor neurological deficits initially experienced neurological deterioration during the acute period, and ~30% of patients presenting with mild symptoms showed unfavorable functional outcomes (11, 12). Therefore, there is a need to identify factors associated with these outcomes in patients with these gray zones.

Stroke is associated with critical disabilities in humans, and the administration of alteplase is the only medical treatment for the hyperacute phase. Therefore, re-evaluation of indications is necessary, and the analysis of factors associated with poor functional outcomes among patients excluded from IV alteplase can help to modify the exclusion criteria. The objective of this study was to investigate factors associated with functional disabilities at 90 days in patients excluded from alteplase administration due to the initial NIHSS scores of 0–5 and minor non-disabling neurological deficits.

## 2. Methods

The local Institutional Review Board reviewed the study. It waived the requirement for ethical approval in compliance with governmental laws and regulations and informed consent since we only accessed de-identified previously collected data.

### 2.1. Study population

**Figure 1:**
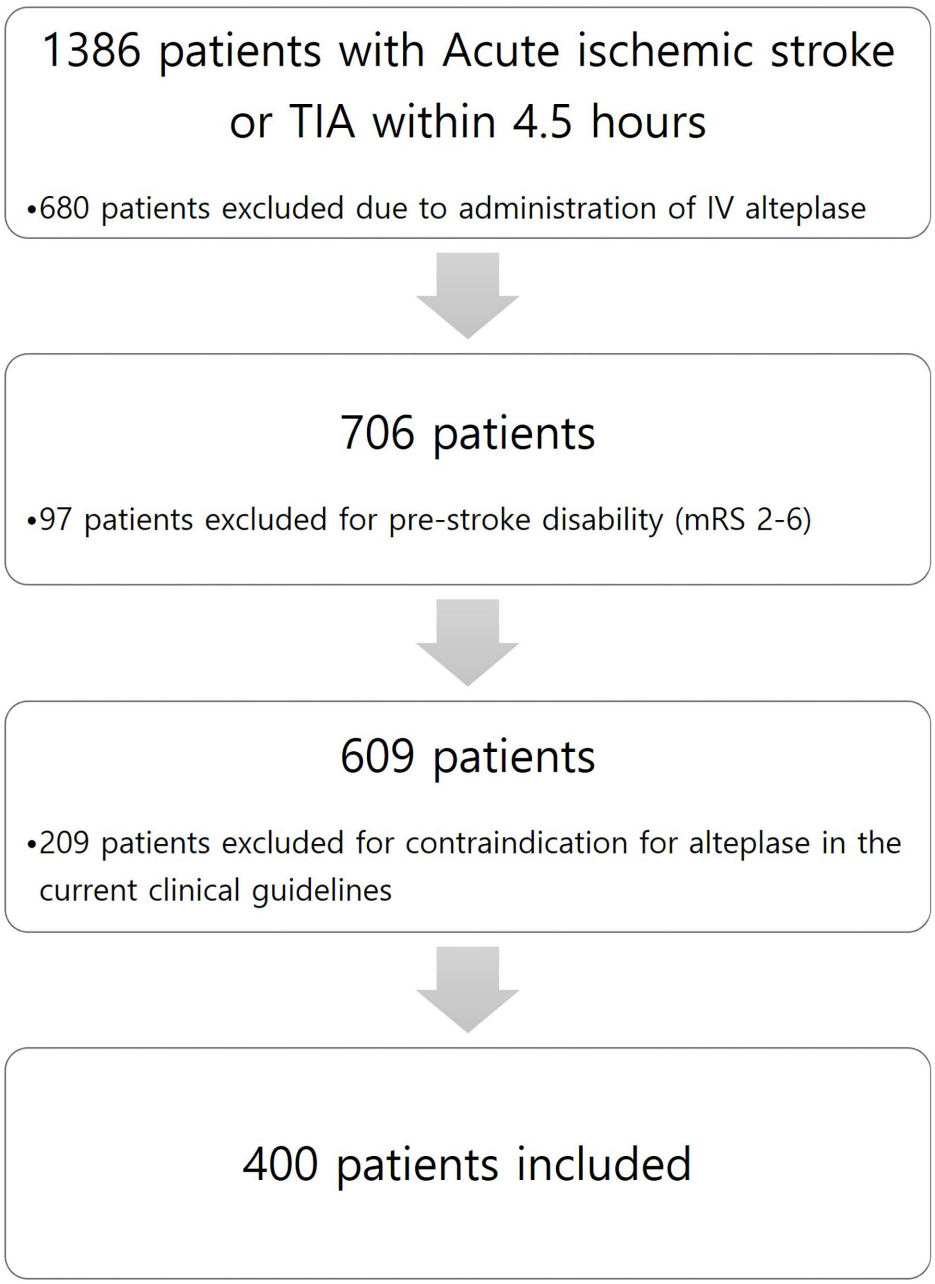
Patient flow of this study.

This study is a retrospective observational study conducted in a single center. Among all patients diagnosed with acute ischemic stroke or TIA within 4.5 h of admission to a tertiary hospital from 1 January 2013 to 31 December 2020, we enrolled patients with acute ischemic stroke who did not receive alteplase due to NIHSS scores of 0–5 and whose deficits were not initially disabling. Minor non-disabling acute ischemic stroke was identified as patients with baseline National Institutes of Health Stroke Scale (NIHSS) score ≤ 5 and a score between 0 and 2 for each NIHSS score item. We excluded patients with pre-stroke disability (modified Rankin Scale score of 2–6) and with contraindication for alteplase in the current clinical guidelines. Figure 1 shows patient flow according to inclusion and exclusion criteria. Patients who did not receive alteplase received 100 mg of oral aspirin and 75 mg of clopidogrel. According to our local protocol, patients with acute ischemic stroke or TIA within 4.5 h underwent baseline neuroimaging, such as precontrast brain computer tomography (CT), brain CT angiography, and brain magnetic resonance image (MRI), including diffusion restriction image and fluid-attenuated inversion recovery (FLAIR). In addition, all patients who were hospitalized and received dual antiplatelet therapy were checked for drug resistance tests by using a new-generation impedance aggregometer (Multiplate^R^ analyzer; Roche Diagnostics, Mannheim, Germany). Clinical management was based on institutional protocols and clinical guidelines for stroke care. The patient underwent follow-up neuroimaging (MRI) within 20–28 h after symptom onset.

### 2.2. Data collection and methods

Radiological data and clinical outcomes that were collected included: (1) baseline neuroimaging, NIHSS score, and mRS; (2) repeat neuroimaging after 20–28 h; (3) discharge NIHSS score, TOAST classification (consisting of small artery disease [SAD], large artery disease [LAD], cardioembolic [CE], other etiology [others], and undetermined etiology [Undetermined]), and mRS; and (4) mRS after 90 days, scored during outpatient follow-up. To compare minor non-disabling stroke presenting as mild dysarthria, ataxia, and mild motor weakness, we defined moderate motor weakness (including dysarthria and facial weakness) when the limb motor score of the NIHSS was ≥3. Early neurological deterioration (END) was defined as an increase of two or more NIHSS scores compared with the best neurological status within 7 days after stroke (13). We defined favorable functional outcomes at 90 days after stroke as a modified Rankin Scale (mRS) score of 0 or 1 (total range, 0 [symptom-free] to 6 [dead]). Fluid-attenuated inversion recovery hyperintense arteries (FLAIR-HAs) were defined as focal, tubular, or serpentine hyperintensities that correspond to a typical arterial course in the FLAIR sequence (14).

### 2.3. Statistical analyses

Categorical variables were expressed as numbers and percentages, while continuous variables were expressed as mean ± standard deviation. An analysis was performed to determine whether the etiology of cerebral infarction according to TOAST classification and factors of anterior and posterior circulation infarction affected the clinical outcome and frequency of END. Multiple regression analysis was performed to determine predictive factors for poor functional outcomes in patients who did not receive alteplase 90 days after stroke. Potential predictors (*P* ≤ 0.2) in the univariate analysis were included in the full multivariate model. The final multivariate model was adjusted for sex. The study rejected the null hypotheses (no difference between the groups) if *P*-values were < 0.05 and considered equivalence if the 95% confidence intervals (CIs) of risk point estimates excluded 1. Statistical Package for Social Sciences 19.0 (SPSS Inc., Chicago, Illinois, USA) was used for statistical analyses.

## 3. Results

### 3.1. Characteristics of the study population

A total of 400 patients who were not administered alteplase [alteplase (–) group] were enrolled from 1 January 2013 to 31 December 2020, with a mean age of 64.8 ± 12.3 years; 246 (61.5%) of these were women. The baseline characteristics of patients are shown in Table 1. The medical risk factors of the study group were as follows: hypertension, 223 patients (55.8%); diabetes mellitus, 113 (28.3%); hyperlipidemia, 66 (16.5%); smoking, 205 (35%); and atrial fibrillation, 62 (15.5%). Notably, 57 (14.3%) and 40 (10.0%) patients had histories of stroke and coronary heart disease, respectively. The most prevalent stroke subtypes were: SAD in 127 patients (31.8%), LAD in 118 (29.5%), and CE in 86 (21.5%). The most prevalent baseline NIHSS was 0 (36.3%) in the alteplase (–) group.

**Table 1:** Baseline characteristics of this study.

	**Intravenous alteplase (–)** ** (*n* = 400)**
Age, years	64.8 ± 12.3
Male, *n* (%)	246 (61.5)
**Risk factors**, ***n*** **(%)**	
Hypertension	223 (55.8)
Diabetes mellitus	113 (28.3)
Atrial fibrillation	62 (15.5)
Hyperlipidemia	66 (16.5)
Cardiac disease	40 (10.0)
Previous stroke	57 (14.3)
Smoking	149 (37.3)
**Baseline laboratory findings**	
Hemoglobin, g/dl	13.9 ± 1.7
Glucose, mg/dl	134.3 ± 49.7
Total cholesterol, mg/dl	174.3 ± 39.9
CRP, mg/dl	0.51 ± 1.69
Uric acid, mg/dl	5.5 ± 1.6
Transient ischemic attack, *n* (%)	105 (26.3)
**Location of Stroke**, ***n*** **(%)**	
Anterior circulation	273 (68.3)
Posterior circulation	115 (28.8)
MR negative	12 (3.0)
**Stroke subtype**, ***n*** **(%)**	
LAD	118 (29.5)
Intracranial stenosis	74 (67.7)
Extracranial stenosis	44 (37.3)
SAD	127 (31.8)
CE	86 (21.5)
Others	22 (5.5)
Undetermined	47 (11.8)
**Baseline NIHSS**, ***n*** **(%)**	
0	145 (36.3)
1	122 (30.5)
2	65 (16.3)
3	32 (8.0)
4	16 (4.0)
5	20 (5.0)
mRS score of 0 or 1 at 90 days	311 (77.8)

LAD, large artery disease; SAD, small artery disease; CE, cardioembolism; others, cryptogenic embolism and hematological coagulopathy; NIHSS, NIH Stroke Scale; MR, magnetic resonance.

### 3.2. Analysis for predicting poor functional outcomes at 90 days in the alteplase (–) group

There was no significant relationship in aspects of functional outcome at 3 months in the factors of anterior and posterior circulatory infarction. LAD was significantly more prevalent (42.7%, *P* = 0.042) than other TOAST classifications in 89 patients with poor functional outcomes (2–6 mRS at 90 days) (Table 2).

**Table 2:** Analysis of functional outcome according to TOAST classification in intravenous alteplase (–) group.

**Outcomes**	**mRS 0–1** ** (*n* = 311)**	**mRS 2–6** ** (*n* = 89)**	***P***
Stroke subtype, *n* (%)			0.042
LAD	80 (25.7)	38 (42.7)	
SAD	104 (33.4)	23 (25.8)	
CE	71 (22.8)	15 (16.9)	
Others	17 (5.5)	5 (5.6)	
Undetermined	39 (12.5)	8 (9.0)	

LAD, large artery disease; SAD, small artery disease; CE, cardioembolism; others, cryptogenic embolism and hematological coagulopathy; NIHSS, NIH Stroke Scale.

In the alteplase (–) group, END occurred significantly more frequently in the LAD group than in the non-LAD TOAST classification (11.0 vs. 5.3%; *P* = 0.042, Table 3). However, a factor of intracranial and extracranial atherosclerotic stenosis was not significantly related to END and poor functional outcomes at 90 days. Although the univariate regression analysis demonstrated that age per 1-year increase, NIHSS per 1-point increase, FLAIR-HAs, presenting as moderate motor weakness, and LAD were significant predictors for poor functional outcomes, multivariate regression analysis revealed that NIHSS per 1-point increase, presenting as moderate motor weakness, and LAD were independent predictors for poor functional outcomes (OR, 1.811 NIHSS per 1-point increase; 95% CI 1.503–2.182; *P* < 0.0001; OR, 2.173 presenting as moderate motor weakness; 95% CI 1.028–4.595; *P* = 0.042; OR, 2.033 LAD; 95% CI 1.099–3.762; *P* = 0.024, respectively) (Table 4).

**Table 3:** Correlation between large artery disease and early neurological deterioration in intravenous alteplase (–) group.

**Outcomes**	**LAD** ** (*N* = 118)**	**Non-LAD** ** (*N* = 282)**	***P***
Early neurological deterioration (+)	13 (11.0)	15 (5.3)	0.042
Early neurological deterioration (–)	105 (89.0)	267 (94.7)	

LAD, large artery disease.

**Table 4:** Multivariate analysis of prediction for poor functional outcome.

	**Estimated ORs for poor functional outcome**			

**Model 1**	**Crude (95% CI)**	***p***	**Adjusted (95% CI)**	***p***
Age, per 1-year increase	1.034 (1.012–1.057)	0.002	–	–
NIHSS, per 1-point increase	1.755 (1.455–2.117)	< 0.0001	1.811 (1.503–2.182)	< 0.0001
Atrial fibrillation	1.413 (0.752–2.653)	0.283	–	–
Hypertension	1.041 (0.635–1.705)	0.874	–	–
Diabetes mellitus	1.552 (0.935–2.574)	0.089		
Cardiac disease	1.675 (0.818–3.431)	0.159		
Previous stroke	1.373 (0.725–2.603)	0.331		
Smoking	0.783 (0.473–1.297)	0.342		
Hyperlipidemia	0.476 (0.217–1.043)	0.064	–	–
Female	1.193 (0.726–1.962)	0.486	–	–
FLAIR-HAs	1.910 (1.133–3.218)	0.015	–	–
Moderate motor weakness	2.667 (1.391–5.111)	0.003	2.173 (1.028–4.595)	0.042
LAD	2.115 (1.270–3.522)	0.004	2.033 (1.099–3.762)	0.024

FLAIR-HAs, fluid-attenuated inversion recovery hyperintense arteries; LAD, large artery disease.

## 4. Discussion

In this retrospective study, 77.8% of patients with minor, non-disabling acute ischemic stroke that did not receive IV alteplase showed favorable functional outcomes at 90 days. Our study found that NIHSS per 1-point increase, presenting as moderate motor weakness, and LAD were independent predictors of poor functional outcomes at 90 days in patients with acute ischemic stroke who did not receive alteplase due to an NIHSS score of 0–5.

Our study showed that moderate motor weakness (limb motor NIHSS score ≥3) was significantly associated with poor functional outcomes at 90 days compared with minor deficits, including mild dysarthria, ataxia, and mild motor weakness. Choi et al. reported that all 15 items of the NIHSS, except sensory and extinction items, were significantly associated with unfavorable functional outcomes and that using the total NIHSS score effectively predicted functional outcomes of mild stroke (15). However, Fischer et al. reported that a minor stroke defined as a score ≤ 1 on every NIHSS item or NHISS ≤ 3 showed favorable outcomes and would be suited to the definition of minor stroke (16). Among his definitions of a minor stroke, the definition of only motor deficits, including dysarthria or ataxia with or without sensory deficit, showed a more significant disability at 3 months. This definition was less suitable for defining a minor stroke. Our findings suggest that patients with minor stroke presenting with moderate motor weakness should be cautiously considered for alteplase administration.

In this study, predictors for poor functional outcomes at 90 days were NIHSS per 1-point increase and LAD in patients who did not receive alteplase due to minor, non-disabling acute ischemic stroke within 4.5 h. There were only two drug resistance (one for aspirin and one for clopidogrel) in 28 END patients; it seems that drug resistance did not affect END. In patients with poor functional outcomes at 90 days despite presenting with minor and non-disabling deficits, the LAD subtype was found to be significantly more prevalent compared with other TOAST classifications. END occurred significantly more frequently in the LAD group than in the non-LAD TOAST group. We believe that the poor functional outcomes in the LAD group can be explained by the more frequent END associated with LAD. In the multivariate analysis, higher baseline NIHSS and LAD were predictors of poor functional outcomes. Sato et al. reported that patients who have large vessel occlusive lesions were 2.80 times more prone to unfavorable functional outcomes at 3 months among acute ischemic stroke patients presenting with NIHSS <3. In addition, Kim et al. showed that END occurred in 14.6% of patients with minor stroke (<3 NIHSS) within 6 h. The only predictor for neurological progression was large vessel occlusion (11, 17). Our results correlate well with those of previous studies. LAD may be an important factor for predicting functional outcomes and should be considered an indicator of alteplase administration in patients with minor stroke within 4.5 h.

Recently, studies on early-warning blood biomarkers and imaging markers in hyperacute cerebral infarction have been actively conducted. The plasma neurofilament light chain is being studied as an index suggesting the possibility of END in patients with acute cerebral infarction (18). Zhou et al. reported that five metabolic markers, such as sphingomyelin (18:0/14:0), 1-methylpyrrolinium, phosphatidylcholine (18:0/18:0), lysophosphatidylcholine (18:0/0:0), and phosphatidylcholine (18:2/18:2), have good diagnostic and predictive ability. The change level of these metabolites is significantly related to ischemic stroke and provides early warning for the diagnosis of atherosclerosis-induced ischemic stroke (19). In acute ischemic stroke patients with severe intracranial arterial stenosis or occlusion, the asymmetrical prominent cortical vein sign might be a useful neuroimaging marker for predicting END (20). In addition, low FLAIR vascular hyperintensity ASPECTS is associated with a higher risk of END in patients who are receiving DAPT (21). Despite these various studies and attempts looking for markers of predicting END, there is no clinically proven predictor for END yet.

Although there is no objective evidence of the benefit of using alteplase in patients who present with an NIHSS score of 5 or less and disabling disability, ~30% of patients presenting with mild ischemic stroke showed unfavorable functional outcomes at 90 days. There may be a gray zone in patients with minor ischemic stroke contraindicated for IV alteplase administration. In our study, although FLAIR-HAs were not significant in the multivariate analysis, they were a significant predictor of poor functional outcomes in the univariate analysis. The FLAIR-HAs result from retrograde flow through the collateral arterial circulation and are related to the presence of large vessel stenosis or occlusion (22). The results of our study suggested that patients with large vessel occlusive disease should be considered as candidates for IV alteplase treatment even though presenting as minor stroke, and the LAD patient group should be defined into detailed subclassification according to various imaging parameters, such as the ratio of the penumbra to the infarct core in perfusion imaging studies, FLAIR-HAs in MR images, or diffusion–perfusion mismatch ratios. Therefore, a large-scale and well-organized study is needed to define certain imaging-based criteria for alteplase administration in patients presenting with minor and non-disabling ischemic stroke.

This study had several limitations. First, it was conducted retrospectively. Second, the number of enrolled patients was relatively small. Finally, because this study was conducted in a medical center, the results need to be carefully interpreted.

There is no concrete evidence for IV thrombolysis in patients with acute ischemic stroke presenting with mild neurological deficits. However, patients presenting with moderate motor weakness or large-vessel occlusive disease showed significantly higher unfavorable functional outcomes at 90 days. Therefore, aggressive treatment with IV thrombolytic agents may be considered cautiously in acute stroke presenting as moderate motor weakness or LAD, even if the neurological deficits are mild.

## Data availability statement

The raw data supporting the conclusions of this article will be made available by the authors, without undue reservation.

## Ethics statement

Ethical review and approval was not required for the study on human participants in accordance with the local legislation and institutional requirements. Written informed consent for participation was not required for this study in accordance with the national legislation and the institutional requirements.

## Author contributions

YK participated in data analysis and writing of the manuscript. SC participated in data collection and data analysis. TK, H-MP, and DJS participated in data collection. DHS acquired the funds, designed the study, involved in data analysis, writing of the manuscript, and supervised the project. All authors read and approved the final version of the manuscript.
